# Characteristics of Communication Competency in Nurse Managers: A Systematic Review

**DOI:** 10.3390/healthcare14172814

**Published:** 2026-09-02

**Authors:** Alberto González-García, Pilar Marqués-Sánchez, Cristian Martín-Vázquez, Gema Serrano-Gemes, David Bermejo-Martínez, Silvia Pérez-González

**Affiliations:** 1 HeQoL Research Group, Department of Nursing and Physiotherapy, León Biomedical Research Institute (IBIOLEÓN), Universidad de León, Cuidados Enfermeros Para la Calidad de Vida Relacionada con la Salud (CECAVIS), 24071 León, Spain; agong@unileon.es; 2 SALBIS Research Group, Department of Nursing and Physiotherapy, León Biomedical Research Institute (IBIOLEÓN), Universidad de León, Campus Ponferrada, Cuidados Enfermeros Para la Calidad de Vida Relacionada con la Salud (CECAVIS), 24401 Ponferrada, Spain; pilar.marques@unileon.es; 3 HeQoL Research Group, Department of Nursing and Physiotherapy, León Biomedical Research Institute (IBIOLEÓN), Universidad de León, Campus Ponferrada, Cuidados Enfermeros Para la Calidad de Vida Relacionada con la Salud (CECAVIS), 24401 Ponferrada, Spain; cmartv@unileon.es; 4 SALBIS Research Group, Department of Nursing and Physiotherapy, Universidad de León, Campus Ponferrada, 24401 Ponferrada, Spain; gserg@unileon.es; 5 SALBIS Research Group, Department of Nursing and Physiotherapy, Universidad de León, 24071 León, Spain; 6 SALBIS Research Group, Cuidados Enfermeros Para la Calidad de Vida Relacionada con la Salud (CECAVIS), León Biomedical Research Institute (IBIOLEÓN), Hospital de León, Complejo Asistencial Universitario de León, 24008 León, Spain; sperg@unileon.es

**Keywords:** nurse managers, communication competency, competency characteristics, nursing leadership, nursing management, healthcare organizations

## Abstract

**Highlights:**

This systematic review identifies the defining characteristics of communication competency in nurse managers and clarifies its relevance as a core managerial capability in healthcare organizations. The findings show that this competency is structured around active listening, strategic communication planning, trust-building, transparency, and message clarity, and that its expression depends on organizational context, leadership behaviors, and healthcare complexity.

**What are the main findings?**

- Communication competency in nurse managers is composed of 23 defining characteristics, with active listening, strategic multi-channel communication, trust-based relationships, transparency, and message clarity emerging as the most frequently reported elements.
- Communication competency is context-dependent and expressed through relational, strategic, behavioral, and organizational dimensions, including crisis communication, informal networks, staff buy-in, rural settings, alternative work arrangements, and digitally complex environments.

**What are the implications of the main findings?**

- The identified characteristics provide a practical basis for designing competency-based leadership training, simulation-based learning activities, communication assessment tools, and professional development programs for nurse managers.
- Strengthening nurse managers’ communication competency may improve psychological safety, team cohesion, staff engagement, workforce retention, and the perceived quality and safety of care in increasingly complex healthcare organizations.

**Abstract:**

**Background:** Communication competence is essential for nurse managers because it facilitates team coordination, organizational alignment, shared decision-making, and the quality and safety of care. Despite its acknowledged importance, the key traits of this competence have not been systematically summarized. **Objective:** We aimed to identify and describe the traits of communication competence among nurse managers in healthcare settings, determine the most frequently reported traits, and explore the contextual, behavioral, and organizational factors associated with its expression. **Methods:** A systematic review was conducted in accordance with PRISMA guidelines and registered with PROSPERO (CRD420261356065). Searches were performed in Web of Science, Scopus, PubMed, and CINAHL, covering 1 January 2016 to 1 February 2026. Peer-reviewed studies using quantitative, qualitative, or mixed methods that addressed communication competence from the perspective of nurse managers were included. Data analysis involved a structured narrative synthesis that combined frequency analysis of identified traits with contextual analysis of communication competence. **Results:** Fifteen studies met the inclusion criteria. After comparison, grouping, and conceptual refinement, 23 communication traits were identified. The most frequently reported traits were Active Listening and Verification of Understanding, along with Strategic and Multi-Channel Communication Planning (each 10.14%), followed by Building Trust-Based Professional Relationships (9.18%), Open and Transparent Communication (8.21%), and Clear, Consistent, and Effective Message Design (7.73%). The results also indicated that communication competence is context-dependent and linked to organizational factors such as structural empowerment, shared decision-making, and managerial visibility. Empowering communication behaviors correlated with psychological empowerment, team cohesion, staff retention, and perceived improvements in quality of care. Conversely, limiting behaviors were associated with declines in trust, dignity, and professional functioning. **Conclusions:** Communication competence among nurse managers is not merely a peripheral interpersonal trait but a core managerial skill with a well-defined foundation. Its key elements suggest that this competence is built on responsiveness, strategic intentionality, and relational credibility. Therefore, communication competence should be viewed as an organizationally significant capability that directly affects team performance, workforce sustainability, psychological safety, and the quality of care. Implications for Nursing Management: Recognizing a core set of communication traits provides a useful basis for leadership training, simulation exercises, and managerial evaluations. Improving this skill could strengthen team unity, boost staff involvement, and enhance the quality of care as healthcare settings become more complex.

## 1. Introduction

Healthcare systems are increasingly complex organizational environments, characterized by continuous technological change, rising care demands, interprofessional interdependence, and the need to respond efficiently to uncertain and rapidly evolving clinical situations [1]. In such settings, organizational performance depends not only on the availability of resources or technical expertise but also on the quality of coordination, information flow, and professional interaction across teams and hierarchical levels [2]. As healthcare delivery becomes more dynamic and multidimensional, communication emerges as a fundamental condition for maintaining organizational coherence, supporting collaboration, and sustaining safe and effective care [3].

Accordingly, nurse managers occupy a pivotal position within healthcare organizations, functioning at the intersection of clinical care, team coordination, and organizational management [4]. Their role extends beyond supervising nursing staff and includes planning and organizing care delivery, coordinating human and material resources, supporting teamwork, evaluating outcomes, and contributing to the achievement of institutional objectives [5]. In this sense, nurse managers serve as a critical link between strategic priorities and daily clinical practice, directly influencing the work environment, professional performance, and the quality and safety of care [6].

Therefore, effective performance in nurse management cannot be explained solely by formal position, years of experience, or administrative responsibility [4,7]. Rather, it depends on the development and integration of specific competencies that enable nurse managers to respond appropriately to the clinical, relational, and organizational complexity of healthcare settings [8,9]. In this context, competency may be understood as a multidimensional construct that encompasses knowledge, skills, attitudes, and observable behaviors, enabling professionals to act effectively, adaptively, and consistently in demanding managerial situations [9]. From this perspective, the study of competencies provides a more precise framework for understanding how nurse managers perform their role and what capacities are required to sustain effective leadership and organizational functioning [7,10].

Within this competency-based perspective, a competency should not be understood as a broad or abstract label alone but as a structured set of defining characteristics that give it substantive meaning in practice [9,11]. These characteristics represent the specific elements through which a competency can be identified, described, and differentiated, and they provide the basis for its conceptual clarification and operationalization [12]. In this sense, analyzing the characteristics of a competency is essential because it enables the translation of a general managerial domain into observable, interpretable components that can be assessed, developed, and incorporated into training processes [9]. Accordingly, the identification of characteristics is not a secondary descriptive exercise but a necessary step for understanding the internal structure of a competency and for supporting its evaluation and professional development in nurse management [7,11,12].

From this perspective, communication emerges as one of the most important competencies in nurse management, as it underpins how managers coordinate teams, align organizational and clinical priorities, support shared decision-making, manage conflict, provide professional guidance, and sustain collaboration across disciplines and hierarchical levels [13,14]. Its relevance is particularly evident in healthcare environments marked by constant change, high relational demands, and increasing organizational complexity, where ineffective communication may compromise team functioning, adaptation processes, and patient safety [7,15]. Accordingly, communication should be understood not merely as a supportive managerial skill but as a core competency through which nurse managers influence professional interaction, organizational coherence, and the quality of care [16].

At the same time, communication in nurse management cannot be reduced to the simple transmission of information or the efficient delivery of instructions [17]. Contemporary healthcare organizations require a more complex understanding of communication as a relational, strategic, adaptive, and context-sensitive competency through which nurse managers interpret situations, build trust, foster dialogue, regulate interactions, and adjust messages, channels, and timing to meet organizational demands [18]. In this sense, effective communication is not defined solely by the ability to speak clearly but also by the capacity to listen actively, foster mutual understanding, anticipate communicative needs, and sustain coordinated action under conditions of uncertainty and change [19].

Although communication is widely recognized as important in nursing management, previous reviews have examined nurse manager competencies from a broad perspective [9,20] or have addressed nurse manager communication primarily in relation to professional and organizational outcomes [15]. However, these reviews did not conceptualize communication as a distinct managerial competency or systematically identify, compare, and organize the characteristics that define its internal structure. To the best of our knowledge, no previous systematic or scoping review has specifically synthesized the defining characteristics of communication competency in nurse managers. Existing studies have generally addressed communication through related constructs, assessment instruments, outcomes, or situational applications [16], without providing an integrated characterization of this competency. Consequently, communication competency in nurse managers remains insufficiently specified at the conceptual level, limiting its precise assessment, targeted development, and incorporation into competency-based managerial training [21]. This gap highlights the need for a synthesis that identifies, describes, and organizes the defining characteristics of communication competency among nurse managers.

Accordingly, in this study, we posed the following research question: What characteristics define communication competence among nurse managers in healthcare settings? To address this research question, the objectives of the present study were as follows:

Objective

- To identify and describe the characteristics of communication competency in nurse managers in healthcare settings.

Specific objectives.

- To determine the most frequently reported characteristics of communication competency in nurse managers.
- To identify contextual, behavioral, and organizational elements associated with communication competency in nurse managers, including communication strategies, antecedents, consequences, and factors influencing its expression.

## 2. Materials and Methods

### 2.1. Design

A systematic review was conducted to identify and describe the characteristics of nurse managers’ communication competency. This review adhered to the Preferred Reporting Items for Systematic Reviews and Meta-Analyses (PRISMA) guidelines [22]. The Joanna Briggs Institute (JBI) guidelines were used to assess the quality of the included articles [23,24,25]. The research question, formulated using the Population–Concept–Context (PCC) framework, was as follows [26]: what are the defining characteristics of communication competency (C) among nurse managers (P) in healthcare settings (C)? The review protocol was registered in PROSPERO (CRD420261356065).

For the purposes of this review, the Population was defined as nurse managers with formal responsibility for supervising nursing personnel, coordinating a clinical unit or service, or performing middle- or senior-level nursing management functions. This definition included head nurses, nurse supervisors, nurse unit managers, nurse administrators, directors of nursing, and nurse executives. Studies were considered eligible whether the information was provided directly by nurse managers or by nursing staff specifically evaluating the communication competency or communication practices of an identifiable nurse manager.

The Concept was communication competency in the nurse manager role, operationally defined as any explicitly reported knowledge, skill, ability, behavior, practice, or assessment dimension concerning how nurse managers receive, interpret, transmit, adapt, or manage information and professional interactions. To be eligible, a study had to provide directly extractable information concerning at least one characteristic of this competency.

The Context comprised healthcare organizations and clinical care settings in which nurse managers performed formal managerial responsibilities, irrespective of the level of care, geographical location, or organizational setting.

A systematic review was selected because the objective was not merely to map the available literature but to identify, critically appraise, and synthesize empirical evidence on the defining characteristics of communication competency. As the research question did not involve an intervention, comparator, or predefined outcome, the PICO framework was not considered suitable. The PCC framework was therefore used solely to define the Population, Concept, and Context of this concept-focused question and did not determine the review design.

This systematic review adapted specific procedural components of the methodological framework established by González-García et al. (2021) [9], namely the search strategy, article selection criteria, and data extraction and coding procedures. This methodology was selected because the current research aligns with the ongoing line of inquiry to implement a competency model for nurse managers, developed by González-García (2019) [27]. Ensuring methodological consistency is crucial for maintaining the coherence of research findings. In addition, this process is registered in the intellectual property registry 00/2024/3056 of the Ministry of Culture in Spain.

### 2.2. Search Strategy

A systematic search was conducted across four electronic databases, namely Web of Science, Scopus, PubMed, and CINAHL, covering the period from 1 January 2016 to 1 February 2026. This timeframe was selected to capture the most recent and methodologically relevant evidence on communication competency among nurse managers while ensuring sufficient conceptual and empirical breadth, given the limited volume and heterogeneity of research in this field. In addition, the selected period encompasses major transformations in healthcare organizations, including increased technological integration, greater organizational complexity, and a growing emphasis on leadership and communication competencies in nursing management. No language restrictions were applied during the search process.

The search strategy was developed in accordance with the methodological approach used in this line of research and combined terms related to nurse management roles with terms associated with communication competency. Nurse management terms included nurse manager, nurse supervisor, nursing program manager, nurse unit manager, chief nurse executive, nurse administrator, director of nursing, head nurse, frontline manager, nursing director, and nursing executive. These were combined with communication-related terms, including communication, communication skills, interpersonal communication, and leadership communication. The full search strategy for each database is presented in File S1, Table S1.

### 2.3. Inclusion and Exclusion Criteria

Original peer-reviewed studies using quantitative, qualitative, or mixed-methods designs were included when they specifically examined communication competency in the nurse manager role and provided extractable information on at least one communication-related characteristic, skill, behavior, practice, or assessment dimension. Eligible studies were published between 1 January 2016 and 1 February 2026. Evidence could be provided directly by nurse managers or by nursing staff specifically evaluating the communication of their nurse managers. Studies involving mixed professional or managerial samples were included only when findings relating specifically to nurse managers could be identified and extracted separately. Studies were excluded when they addressed only nurse–patient communication, communication among staff nurses, general organizational communication, or interprofessional communication without providing separately extractable information concerning nurse managers.

Review articles, doctoral dissertations, editorials, letters to the editor, opinion papers, conference proceedings, and other forms of grey literature were excluded. This approach was adopted to prioritize primary, peer-reviewed evidence directly relevant to the review’s objective and to maintain consistency with the research framework used to operationalize the competency model for nurse managers. Although this decision may have reduced the breadth of available evidence, it strengthened the review’s methodological consistency by focusing on primary peer-reviewed studies.

### 2.4. Study Selection

The selection process entailed three main steps: (1) the search results were initially exported to Mendeley for reference management and subsequently transferred to Covidence to support systematic screening and study selection; (2) duplicates and non-relevant articles were removed through a thorough screening of titles and abstracts; and (3) articles were excluded in accordance with the established eligibility criteria. The screening of titles, abstracts, and full texts for each retrieved article was conducted independently by two researchers (A.G.-G and P.M.-S). Any disagreements between the researchers were resolved through discussion and, if necessary, further reviewed by consultation with a third reviewer (S.P.-G).

### 2.5. Data Extraction

Before data extraction and synthesis, the research team specified the data items and synthesis domains to be collected using structured forms developed specifically for this review. Two levels of information were defined. First, descriptive and methodological study data were collected, including author and year of publication, country, study design, methodological approach, participants, variables examined, main findings, implications for nurse managers, reported limitations, and information required for methodological quality appraisal.

Second, the conceptual information to be synthesized was specified in accordance with the review objectives. The primary review outcome was the identification and conceptual organization of the defining characteristics of communication competency in nurse managers. The secondary review outcomes comprised: (1) theoretical models, conceptual frameworks, and assessment instruments; (2) communication strategies and skills applied to specific managerial or organizational contexts; (3) antecedents and consequences of communication competency; (4) empowering and limiting communication behaviors; (5) moderators, barriers, and facilitators; and (6) developmental phases associated with the acquisition and consolidation of communication competency throughout the managerial career. In this qualitative narrative systematic review, these outcomes represented the predefined conceptual domains to be extracted and synthesized rather than intervention-related clinical outcomes.

Data extraction was performed independently by two reviewers (A.G.-G. and P.M.-S.) using the same structured extraction form. Once the independent extraction was completed, the two forms were compared to identify discrepancies. Disagreements were resolved through discussion and consensus, with consultation of a third reviewer (S.P.-G.) when required. Study authors were not contacted for additional or missing information; consequently, the review relied exclusively on information available in the published reports.

All included studies were read in full and reviewed repeatedly to ensure comprehensive identification of relevant information. All reported results relating to the primary or secondary review outcomes were extracted. When an included study reported more than one relevant finding, each finding was extracted separately. No result was excluded on the basis of its direction, statistical significance, frequency, or inconsistency with findings from other studies. Information was not retained for synthesis when it was unrelated to communication competency, concerned general leadership or managerial functioning without an explicit communication component, addressed other forms of communication without separately identifiable information concerning nurse managers, or duplicated a finding already extracted from the same study.

To ensure systematic organization and traceability, a coding procedure was implemented. Each article was assigned a unique identifier beginning with the letter “A” followed by three digits, starting with A001. Each extracted communication characteristic was coded as “C” followed by three digits, starting with C001. Accordingly, each unit of analysis could be directly linked to its source. For example, A003 C007 referred to the seventh communication-related characteristic extracted from the third included study.

Because a single article could contribute more than one communication characteristic, frequencies and percentages were calculated using the 207 extracted characteristics as the denominator. Accordingly, these values represent the frequency of the extracted characteristics and not the proportion of included studies.

### 2.6. Review Outcomes

For the purposes of this qualitative narrative systematic review, review outcomes were understood as the concepts to be synthesized rather than as intervention effects. The primary review outcome was the identification, conceptual organization, and description of the defining characteristics of nurse managers’ communication competency, including their frequencies within the complete dataset of extracted characteristics. Secondary review outcomes were: (1) conceptual models, theoretical frameworks, and assessment tools used to examine communication competency; (2) communication strategies and skills applied to specific organizational or managerial contexts; (3) antecedents and consequences of communication competency; (4) empowering and limiting communication behaviors; (5) moderating factors, barriers, and facilitators; and (6) developmental phases associated with the acquisition and consolidation of communication competency throughout the managerial career.

### 2.7. Data Analysis and Synthesis

The heterogeneity of the included studies in terms of design, methodology, variables examined, and descriptions of communication-related characteristics made statistical pooling inappropriate. Therefore, a structured narrative synthesis was undertaken, combining a frequency analysis of the identified characteristics with a contextual analysis of communication competency in nurse managers.

The included studies were examined in depth to synthesize the following information: (1) author and year of publication; (2) study design; (3) variables examined; (4) methods or instruments used to assess communication competency; (5) nurse managers’ approaches to communication; and (6) methodological quality. The Synthesis Without Meta-analysis (SWiM) reporting framework was used to structure the organization and reporting of the synthesis [28]. The analytical procedure comprised four main stages: (1) summarizing the included studies, their principal findings, and their methodological quality; (2) comparing the communication characteristics extracted from all included studies to identify exact or conceptual equivalence and enable their grouping; (3) applying predefined relevance criteria to retain all information directly related to the review objectives and synthesis domains and to exclude information unrelated to nurse managers’ communication competency; and (4) synthesizing the retained evidence coherently to generate analytically meaningful findings aligned with the objectives of the review.

The included studies were not grouped according to conceptual similarity. Instead, all studies providing relevant information contributed to the synthesis of communication competency characteristics. After data extraction, A.G.-G. and P.M.-S. independently compared the complete set of coded characteristics and independently proposed their initial grouping.

Characteristics were grouped when they were described using identical or synonymous terms or when, despite differences in wording, they represented the same underlying communicative ability, behavior, function, or managerial purpose. Characteristics were retained as separate categories when they differed in their primary meaning, communicative function, intended purpose, or behavioral expression, or when merging them would have resulted in a loss of conceptual specificity. Characteristics that could not be meaningfully combined with any other characteristic remained as independent categories.

The groupings independently proposed by A.G.-G. and P.M.-S. were subsequently compared. Disagreements were discussed and resolved by consensus. The proposed groupings and their conceptual labels were then reviewed and validated by C.M.-V., G.S.-G., D.B.-M., and S.P.-G. Final decisions were agreed upon by the full research team. The comparison and refinement process was iterative and continued until no further conceptually justified grouping was possible. Throughout this process, the original article and characteristic identifiers were retained to preserve traceability between each final characteristic, the original extracted information, and its source study.

The number of final characteristics was not predetermined. Each of the 207 coded characteristics was assigned to one final category according to the decision rules described above, thereby avoiding double counting. When several coded characteristics shared the same conceptual meaning, their frequencies were combined within the corresponding final characteristic. When a characteristic could not be conceptually combined with another without loss of meaning, it was retained as an independent category. The label assigned to each final characteristic was agreed upon by the research team and formulated to represent the shared meaning of all coded characteristics included within that category. This iterative process resulted in the consolidation of the 207 extracted characteristics into 23 final communication competency characteristics. Frequencies for each final characteristic were calculated by summing the coded characteristics assigned to that category, using the complete dataset of 207 extracted characteristics as the denominator.

In a subsequent analytical phase, the contextual information previously extracted from the included studies was examined to support a broader interpretation of communication competency. This information included the theoretical models and frameworks underpinning the competency; the measurement instruments used; communication strategies and skills applied to specific organizational or contextual challenges; antecedents and consequences of communication competency; empowering and limiting communication behaviors; moderating factors, barriers, and facilitators; and developmental phases associated with the acquisition and consolidation of communication competency throughout the managerial career.

Information was retained for synthesis when it explicitly described: (1) a characteristic, knowledge component, skill, ability, behavior, or practice related to nurse managers’ communication competency; (2) a communication strategy applied to a specific managerial or organizational context; (3) an antecedent, consequence, moderator, barrier, or facilitator of this competency; (4) an empowering or limiting communication behavior; (5) a developmental phase related to the acquisition or consolidation of communication competency; or (6) a theoretical model, conceptual framework, or assessment instrument used to examine this competency. Descriptive and methodological information required to characterize and critically appraise the included studies was also retained.

Information was excluded from the analytic synthesis when it was unrelated to communication competency, referred to general leadership or managerial functioning without an explicit communication component, addressed nurse–patient, staff-nurse, or interprofessional communication without separately identifiable information concerning nurse managers, or duplicated a finding already extracted from the same study. No finding was excluded because of its direction, statistical significance, frequency, or inconsistency with findings from other studies.

A.G.-G. and P.M.-S. independently applied these relevance criteria during data extraction. Any disagreement concerning whether information should be retained or how it should be categorized was resolved through discussion and consensus, with consultation of S.P.-G. when required. The resulting contextual categories were subsequently reviewed and validated by C.M.-V., G.S.-G., D.B.-M., and S.P.-G., and final decisions were agreed upon by the full research team.

All included studies were considered for each of the planned syntheses. A study was assigned to a specific synthesis when it reported directly extractable information corresponding to that analytical domain. Allocation was not determined by study design, direction or statistical significance of the findings, or methodological quality. Because individual studies could provide information relevant to more than one domain, they could contribute to multiple syntheses. All 15 included studies contributed to the primary synthesis of communication competency characteristics. Six studies contributed to the synthesis of context-specific communication strategies, eight to antecedents and consequences, four to empowering and limiting communication behaviors, seven to moderators, barriers, and facilitators, twelve to conceptual models and assessment tools, and one to developmental phases. The exact allocation of each included study to the corresponding synthesis is reported in File S1, Table S7.

### 2.8. Quality Appraisal

Quality appraisal was performed according to study design. The eight-item JBI Critical Appraisal Checklist for Analytical Cross-Sectional Studies was applied to the quantitative studies and to the two methodological or mixed-methods instrument-development studies whose predominant evaluative component was quantitative [16,29]. The ten-item JBI Critical Appraisal Checklist for Qualitative Research was applied to the qualitative studies. The studies assessed using each tool are reported individually in File S1, Tables S3 and S4.

The adherence percentage was calculated by assigning one point to each “Yes” response and zero points to “No” or “Unclear” responses. The number of “Yes” responses was divided by the total number of checklist items and multiplied by 100. No item was classified as “Not Applicable.” These percentages were reported as descriptive summaries of checklist adherence and were not used as numerical thresholds for the overall risk-of-bias classification.

Overall risk of bias was determined through a qualitative, domain-based assessment rather than by applying numerical thresholds to the checklist adherence percentages. Before assigning the overall risk-of-bias classifications, the following decision rules were established: studies were classified as low risk when no major methodological concern was considered likely to affect the credibility of the findings; moderate risk when one or more relevant methodological concerns could influence interpretation without invalidating the principal findings; and high risk when a critical methodological flaw or multiple serious concerns were considered likely to substantially compromise the credibility of the findings. The assessment considered sampling and participant selection, response rate, sample adequacy, measurement validity and reliability, control of confounding, appropriateness of the analysis, and, where applicable, qualitative methodological congruity and credibility procedures.

Two reviewers (A.G.-G. and P.M.-S.) independently applied the corresponding JBI checklist and assigned the overall risk-of-bias classification to each study. Disagreements were resolved through discussion and consensus, with consultation of a third reviewer (S.P.-G.) when required. A qualitative sensitivity analysis excluding high-risk studies was planned but was not performed because none of the included studies were classified as high risk.

## 3. Results

The findings are presented according to the review outcomes specified in Section 2. The primary review outcome comprises the defining characteristics of nurse managers’ communication competency. The secondary review outcomes provide contextual and interpretive information concerning the models and assessment tools, context-specific communication strategies, antecedents and consequences, empowering and limiting behaviors, moderators, barriers and facilitators, and developmental phases identified across the included studies.

**Figure 1:**
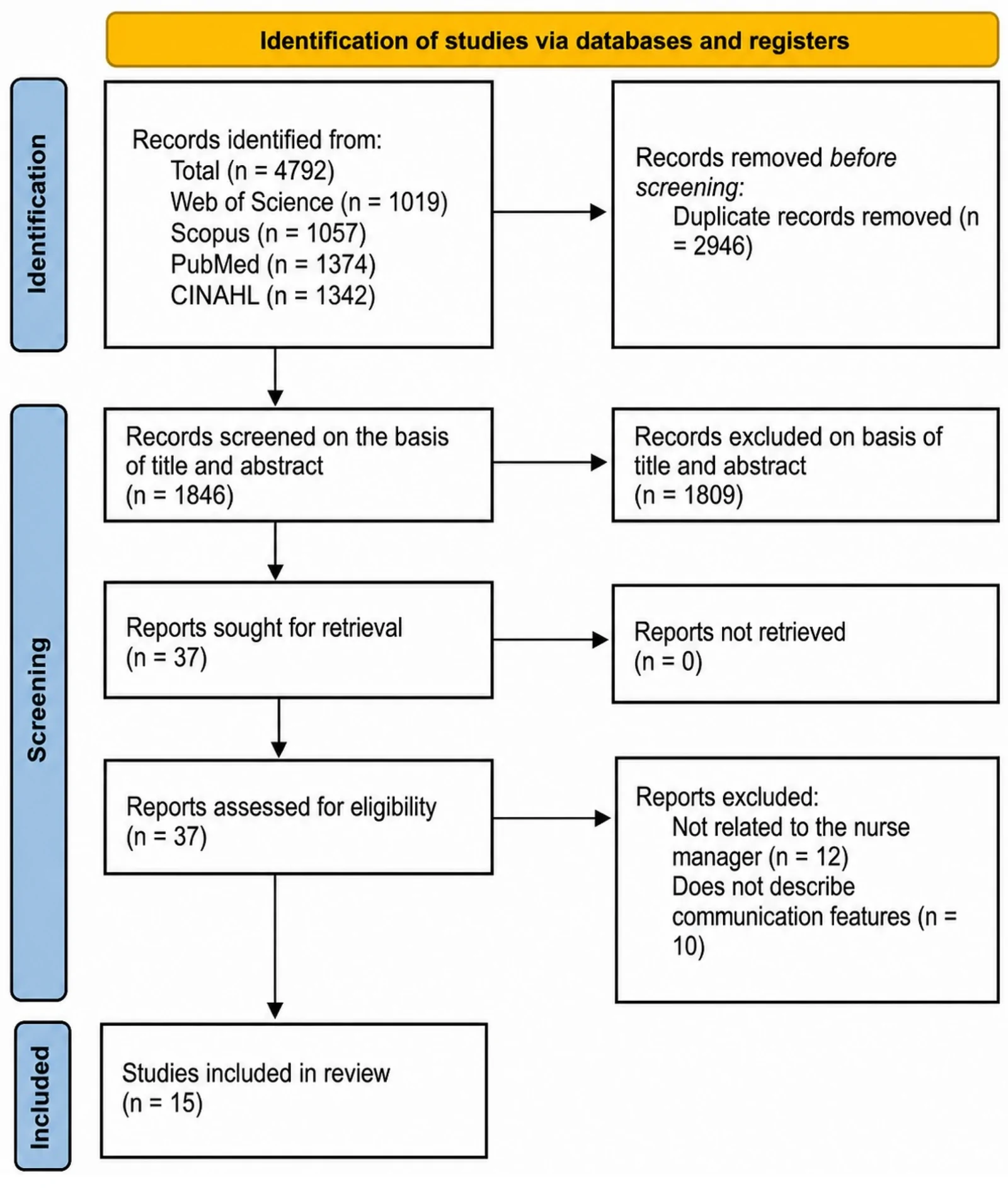
PRISMA flow diagram.

The database search identified a total of 4792 records across the four selected databases. After duplicate removal, 1846 records remained for title and abstract screening. Of these, 1809 were excluded, leaving 37 articles for full-text review. Following application of the eligibility criteria, 22 articles were excluded, and 15 studies were ultimately included in the systematic review. The processes of identification, screening, eligibility assessment, and final inclusion are shown in Figure 1.

The main characteristics of the studies included in this systematic review are presented in File S1, Table S2.

The included studies showed substantial methodological heterogeneity, with a predominance of cross-sectional designs alongside qualitative and methodological studies. A considerable proportion of the studies were conducted in the United States, although evidence also came from Europe, Asia, and the Middle East. Overall, communication competency among nurse managers was consistently associated with key outcomes, including job satisfaction, self-efficacy, team empowerment, buy-in, and relationship quality. Several studies have suggested that this competency extends beyond interpersonal skills to include leadership, ethical sensitivity, contextual adaptation, and digital communication management. In addition, the methodological studies contributed validated instruments that supported a more structured assessment of communication competency.

The results of the quality appraisal ranged from moderate to high. Quantitative and methodological studies reported adherence scores ranging from 50% to 100%, with most demonstrating high adherence to the JBI quality criteria, particularly in clarity of objectives, methodological consistency, and use of appropriate instruments. Lower scores were primarily observed in instrument development studies, in which some methodological domains were only partially addressed (File S1, Table S3).

Qualitative studies also showed generally good methodological quality, with scores ranging from 70% to 100%. The strongest studies demonstrated clear methodological alignment, adequate data collection procedures, and robust strategies to enhance trustworthiness, such as triangulation, member checking, or theoretical saturation. Lower scores were primarily associated with limited reflexivity or partial alignment with some JBI qualitative appraisal domains (File S1, Table S4).

Regarding risk of bias, most studies were classified as having a moderate risk, while a smaller proportion were classified as having a low risk. Specifically, 33.33% of the studies were categorized as low risk and 66.67% as moderate risk. No studies were classified as high risk. Overall, this distribution supports the credibility of the evidence base, although the predominance of moderate-risk studies suggests that the findings should be interpreted with appropriate methodological caution (File S1, Table S5).

From the articles included in this review, 207 characteristics associated with nurse managers’ communication competency were identified. After application of the methodological process of comparison, grouping, and conceptual refinement, 23 distinct characteristics were obtained (Table 1).

The characteristics most frequently cited in the literature were Active Listening and Verification of Understanding and Strategic and Multi-Channel Communication Planning (10.14% each), followed by Building Trust-Based Professional Relationships (9.18%), Open and Transparent Communication (8.21%), and Clear, Consistent, and Effective Message Design (7.73%). These findings underscore the importance of listening, trust-building, openness, and message clarity as core elements of communication competency in nurse managers. In addition, the relevance of feedback, nonverbal communication, and empathic support suggests that effective communication in managerial practice involves not only the transmission of information but also the regulation of relationships, emotions, and team dynamics. The results, therefore, reinforce the need to develop communication training that integrates relational, strategic, and context-sensitive components to prepare nurse managers for complex healthcare environments.

Several conceptual models and assessment tools were identified in the included studies, reflecting the conceptual and methodological diversity with which communication competency in nurse managers has been approached in the literature (File S1, Table S6).

The review identified key communication strategies and skills adapted to specific organizational contexts, which are presented in Table 2.

Several communication strategies and skills are adapted to specific organizational contexts. The most relevant contexts included crises, informal organizational networks, staff buy-in processes, alternative work arrangements, rural settings, and e-leadership environments. Across these contexts, the literature emphasized clear and timely communication, message redundancy, active listening, emotional support, trust-building, and the strategic use of multiple communication channels. These findings show that communication competency in nurse managers is expressed not only through general relational skills but also through context-sensitive strategies adapted to specific organizational demands.

The review also identified key antecedents and consequences of communication competency among nurse managers, as shown in Table 3.

The identified antecedents included communication training, structural empowerment, shared decision-making, managerial visibility, organizational support, and active listening within fair and collaborative work environments. The reported consequences were consistently positive and included higher nurse self-efficacy, greater job satisfaction, psychological empowerment, stronger team cohesion, improved staff retention, and better perceived quality of care. In addition, some studies highlighted broader organizational benefits, such as increased productivity, employee loyalty, and patient-centered care culture. These findings show that communication competency in nurse managers is associated not only with relational and professional benefits but also with relevant organizational and clinical outcomes.

In addition, the review identified empowering and limiting communication behaviors in nurse managers, as shown in Table 4.

The review also identified moderators, barriers, and facilitators that influence the effectiveness of communication among nurse managers, as shown in Table 5.

The identified barriers included psychological distress, message ambiguity, rumors, information overload, geographic isolation, technological limitations, interprofessional hierarchy, and occupational stressors. In contrast, the main facilitators were transparent, formal communication; active listening; empathetic openness; message clarity; multimodal communication channels; and mentoring in digital environments. In addition, factors such as span of control, managerial tenure, psychosocial work conditions, and alternative work arrangements were found to moderate communication effectiveness. These findings indicate that communication competency in nurse managers is shaped not only by individual communication skills but also by organizational, relational, and contextual conditions that may either strengthen or hinder its expression.

## 4. Discussion

This systematic review aimed to identify and describe the characteristics of nurse managers’ communication competency. The findings highlight the current state of research on communication competency in nursing management. Overall, the included studies show that communication competency has been examined through heterogeneous methodological approaches and has been consistently associated with relevant outcomes such as job satisfaction, self-efficacy, empowerment, buy-in, and relationship quality. At the same time, the literature suggests that communication among nurse managers extends beyond basic interpersonal skills and functions as a leadership-related competency shaped by ethical sensitivity, contextual adaptation, and digital demands. These findings are consistent with research on transformational leadership in nursing management, which remains one of the most influential frameworks for understanding effective communication in this context [12,43]. Transformational leadership emphasizes inspirational motivation, individualized consideration, intellectual stimulation, and idealized influence, all of which depend heavily on the manager’s ability to communicate clearly, build trust, listen actively, and adapt messages to the team’s needs. In this review, studies such as those performed by Jankelová et al. (2021) [40], Donohue-Porter et al. (2019) [36], and French-Bravo et al. (2020) [32] suggest that communication competency is closely linked to empowerment, satisfaction, professional commitment, and team engagement. This supports the view that communication is not merely a functional managerial skill but a central mechanism through which nurse managers exert leadership, influence team dynamics, and shape the quality of the work environment [44].

A total of 207 characteristics were identified and analyzed; after applying the methodological processes of comparison, grouping, and conceptual refinement, 23 distinct characteristics were identified. The included studies were also analyzed to contextualize communication competency within the nurse manager role and the healthcare environment. The findings highlight Active Listening and Verification of Understanding and Strategic and Multi-Channel Communication Planning (10.14% each), followed by Building Trust-Based Professional Relationships (9.18%), Open and Transparent Communication (8.21%), and Clear, Consistent, and Effective Message Design (7.73%) as the most salient characteristics of nurse managers’ communication competency. The prominence of active listening supports research emphasizing dialogic, responsive, and person-centered communication as a central leadership resource in complex healthcare settings, where communicative failures may affect both team functioning and the quality of care [45]. Likewise, the relevance of strategic and multi-channel communication planning reinforces evidence that effective nurse managers must not only communicate clearly but also adapt channels, timing, and formats to organizational demands to sustain coordination, reduce uncertainty, and facilitate change [46]. The importance of trust-based relationships, openness, and message clarity further indicates that communication competency is closely linked to the creation of psychologically safe, collaborative, and transparent work environments [47]. In this regard, our findings are consistent with research examining transformational leadership in nursing management, which highlights trust, individualized consideration, open communication, and supportive interaction as key mechanisms for strengthening cohesion, engagement, and professional satisfaction [48].

Beyond these core characteristics, the findings also show that communication competency in nurse managers is highly context-sensitive. The most relevant contexts identified in the literature included crises, informal organizational networks, staff buy-in processes, alternative work arrangements, rural settings, and e-leadership environments. Across these contexts, effective communication was characterized by clarity, timeliness, active listening, emotional support, trust-building, and the strategic use of multiple channels [49,50]. These results add to research suggesting that communication in nursing management cannot be understood solely as a fixed interpersonal skill but rather as an adaptive competency that must be adjusted to organizational demands, uncertainty, and situational complexity [9,16].

From an organizational perspective, the findings further suggest that communication competency in nurse managers is not only an individual professional attribute but also a competency embedded in organizational conditions and associated with measurable clinical and workforce outcomes. The identified antecedents, such as communication training, structural empowerment, shared decision-making, managerial visibility, and organizational support, are consistent with research examining transformational leadership and relational leadership in nursing management, which emphasizes the importance of supportive environments, access to information, and participatory practices for strengthening managerial effectiveness [51,52]. In the same direction, the reported consequences like greater self-efficacy, job satisfaction, psychological empowerment, team cohesion, staff retention, and better perceived quality of care reinforce the idea that communication acts as a mechanism through which nurse managers influence both professional experience and organizational performance [15]. These results also align with research showing that psychologically safe and communicatively open work environments contribute to healthier teams, stronger engagement, and more sustainable care delivery [53].

At the behavioral level, the findings indicate that nurse managers’ communication competency is expressed through both empowering and limiting communicative practices. Empowering behaviors were characterized by active listening, openness, empathy, clear and timely information, constructive private feedback, shared decision-making, and respectful interpersonal treatment. In contrast, limiting behaviors included top-down communication, punitive interaction, public criticism, interruptions, dismissive nonverbal cues, delayed or ambiguous information, favoritism, and aggressive paralanguage. These findings add evidence to research indicating that supportive, respectful, and participatory communication strengthens trust, psychological safety, and team engagement [54,55], whereas demeaning and authoritarian communication undermines cohesion, dignity, and professional commitment [56,57].

Finally, the identification of moderators, barriers, and facilitators further reinforces the view that communication competence among nurse managers is shaped not only by individual capabilities but also by the conditions under which communication occurs. The presence of factors such as psychological distress, rumors, information overload, interprofessional hierarchy, and technological limitations suggests that communication effectiveness is highly sensitive to organizational and contextual pressures. In contrast, transparent communication structures, active listening, empathetic openness, and adequate support mechanisms appear to strengthen the expression of this competency. These findings add to research indicating that communication in nurse management is inseparable from the environments in which it is enacted and sustained [53,58].

This review should be interpreted in light of several limitations. The methodological and conceptual heterogeneity of the included studies precluded meta-analysis. It required a narrative synthesis, which may introduce a degree of interpretive subjectivity despite the structured analytic process applied. In addition, some of the contextual, behavioral, and organizational elements identified were derived from a more limited subset of studies than the core characteristics, which may limit their generalizability. Finally, the predominance of cross-sectional and context-specific evidence may limit the transferability of some findings across organizational and healthcare settings. Nevertheless, the review provides a rigorous, conceptually robust synthesis of the key characteristics that define communication competency in nurse managers.

Future research should move beyond descriptive approaches and examine how nurse managers’ communication competency is developed, enacted, and sustained across different healthcare environments. In particular, longitudinal, cross-cultural, and mixed-methods studies are needed to clarify its developmental trajectory, its impact on workforce and care outcomes, and the organizational conditions that strengthen or constrain its expression. Greater attention should also be given to the design and validation of assessment instruments, simulation-based training models, and implementation strategies that translate the identified core characteristics into observable, transferable managerial practice.

## 5. Conclusions

This review suggests that communication competency in nurse managers is not merely a secondary interpersonal attribute but a core managerial competency that influences how nursing leadership is enacted in healthcare settings. In the analysis of the extracted characteristics, active listening, strategic communication planning, trust-based relationship-building, openness, and message clarity were the characteristics most frequently represented within the extracted dataset. Their greater representation may point to their particular relevance within the conceptualization of communication competency. Together, these five characteristics account for nearly half of the extracted characteristics, suggesting that communication competency may not constitute a diffuse or undifferentiated skill set but rather a structured competency with identifiable areas of emphasis. In practice, this group of characteristics may help nurse managers reduce uncertainty, coordinate teams, promote psychological safety, and contribute to effective organizational and clinical performance.

The fact that active listening, strategic communication planning, and trust-based relationships rank among the characteristics most frequently represented within the extracted dataset is particularly noteworthy. This may indicate that the specialized literature is moving beyond conceptualizing communication in nursing management as merely a transmission function and increasingly understands it as a relational and strategic managerial mechanism. From this perspective, active listening supports psychological safety and legitimizes staff voice, while strategic communication planning helps teams navigate uncertainty, assimilate change, and maintain coordination under pressure. Their greater representation within the extracted dataset reinforces their potential relevance as important areas for the development, training, and assessment of communication competency in nursing management.

Perhaps the most relevant conclusion of this review is that nurse managers’ communication competency should not be understood, developed, or evaluated as an isolated individual attribute. The identification of context-specific strategies, structural antecedents, measurable consequences, and empowering and limiting behavioral patterns suggests that this competency is deeply embedded in the organizational environments in which it is enacted. Empowering communication behaviors—such as active listening, transparent information sharing, constructive private feedback, and shared decision-making—were associated with psychological empowerment, team cohesion, staff retention, and better perceived quality of care. In contrast, limiting behaviors were linked to deterioration in trust, dignity, and professional functioning. These findings may indicate that communication competency is not a secondary managerial priority but a capability directly relevant to workforce sustainability, team performance, and patient safety.

## Figures and Tables

**Table 1:** Clustering and Frequency of Nurse Managers’ Communication Competency Characteristics.

Characteristic	Frequency, n (%)	Description
Active Listening and Verification of Understanding	21 (10.14%)	The ability to listen attentively, without interruption, and with full presence, while acknowledging concerns, paraphrasing meaning, asking appropriate questions, and confirming mutual understanding to support responsive and effective communication.
Strategic and Multi-Channel Communication Planning	21 (10.14%)	The ability to deliberately plan, coordinate, and adapt communication through appropriate channels, formats, and timing, ensuring accessibility, clarity, contextual fit, and effective translation of organizational messages for unit-level practice.
Building Trust-Based Professional Relationships	19 (9.18%)	The ability to establish, sustain, and strengthen professional relationships grounded in trust, respect, accessibility, active listening, and effective collaboration with nursing staff, supervisors, physicians, and informal groups.
Open and Transparent Communication	17 (8.21%)	The ability to foster open, honest, accessible, and two-way communication by creating opportunities for dialogue, sharing relevant information clearly, inviting staff input, and supporting informed participation in decision-making and change.
Clear, Consistent, and Effective Message Design	16 (7.73%)	The ability to formulate clear, coherent, unambiguous, and consistent messages that convey instructions, explanations, and information accurately, logically, and effectively across verbal and organizational communication contexts.
Constructive and Performance-Oriented Feedback	14 (6.76%)	The ability to provide, receive, and sustain timely, private, respectful, and constructive feedback that reinforces strengths, addresses performance concerns, supports improvement, and enables ongoing communicative exchange in professional practice.
Nonverbal and Paralinguistic Communication Regulation	13 (6.28%)	The ability to regulate tone, volume, body language, eye contact, facial expression, and turn-taking in order to convey composure, clarity, respect, and constructive intent during professional interactions.
Empathic and Supportive Communication	12 (5.80%)	The ability to communicate with empathy, emotional regulation, and interpersonal sensitivity by acknowledging feelings, conveying support and respect, managing uncertainty, and responding appropriately to others’ emotional readiness and situational vulnerability.
Adaptive and Audience-Centered Communication	10 (4.83%)	The ability to tailor communication to the needs, characteristics, generational profiles, and contextual conditions of different audiences by adjusting style, feedback, and communicative strategies to specific situations and environments.
Motivational and Persuasive Communication for Buy-In	9 (4.35%)	The ability to use goal-aligned, persuasive, and encouraging communication to gain support, negotiate agreement, elicit positive responses, and mobilize staff toward shared organizational and practice-related objectives.
Communication Self-Awareness and Professional Competence	8 (3.86%)	The ability to understand, apply, and regulate communication knowledge in practice through self-awareness, message exchange competence, accountability, and ongoing self-monitoring during professional interactions.
Coaching, Mentoring, and Staff Development	6 (2.90%)	The ability to support the professional growth of nursing staff through coaching, mentoring, teaching, guidance, and the facilitation of adaptive learning, including the effective integration of technological change.
Collaborative Team Communication	6 (2.90%)	The ability to communicate effectively within the team through information sharing, perspective exchange, cooperative problem-solving, and relational coordination that supports collective understanding and joint action.
Cross-Functional and Hierarchical Communication Awareness	5 (2.42%)	The ability to communicate effectively across professional boundaries and organizational levels, ensuring appropriate liaison, upward and downward information flow, and context-sensitive interaction within multidisciplinary and managerial structures.
Communication Management in Digitally Complex Environments	5 (2.42%)	The ability to manage communication effectively in digitally dense settings by reducing information overload, sustaining clarity across layered virtual exchanges, and identifying, preventing, and correcting rumors and misinformation.
Timely and Accurate Information Dissemination	5 (2.42%)	The ability to communicate precise, current, and relevant information promptly and consistently in order to support patient safety, situational awareness, and staff responsiveness to clinical and organizational developments.
Conflict Prevention and Resolution	5 (2.42%)	The ability to manage tensions, interactions, and emerging disagreements constructively by reducing escalation, preventing adverse responses, and addressing conflict in ways that preserve working relationships and team stability.
Respectful and Equitable Communication	4 (1.93%)	The ability to communicate with fairness, respect, and impartiality by avoiding favoritism, expressing recognition equitably, and ensuring that interpersonal and managerial interactions are perceived as just and balanced.
Composed and Calm Communication	3 (1.45%)	The ability to maintain composure, emotional steadiness, and self-confidence during interactions in order to communicate clearly, preserve relational stability, and respond appropriately under communicative pressure.
Communicative Role Modeling	3 (1.45%)	The ability to exemplify effective communication in practice by serving as a credible role model and by clearly explaining rules, current conditions, and errors to guide staff behavior and professional standards.
Confidentiality Maintenance	2 (0.97%)	The ability to safeguard sensitive information appropriately within professional communication, ensuring discretion, trust preservation, ethical conduct, and respect for privacy in managerial and clinical interactions.
Ethical Communication Sensitivity	2 (0.97%)	The ability to communicate in ways that reflect ethical awareness, moral discernment, and professional responsibility, ensuring that interactions are conducted with integrity, respect, and adherence to ethical communication standards.
Social-Emotional Management	1 (0.48%)	The ability to recognize, regulate, and respond appropriately to emotions within professional interactions in order to preserve relational balance, communicative effectiveness, and emotionally safe care and work environments.

Source: Authors’ own elaboration.

**Table 2:** Communication Strategies and Skills Adapted to Specific Organizational Contexts.

Author & Year	Context/Organizational Challenge	Synthesized Strategies and Key Communication Characteristics
(Khosravani et al., 2018) [30]	Crisis management (e.g., COVID-19 pandemic)	Employing clear, concise, and consistent messaging while restricting content to three or four positive directives. Adhering to the CDC’s Crisis and Emergency Risk Communication (CERC) principles: being first, right, and credible; expressing empathy; promoting action; and demonstrating respect.
(Elsayed et al., 2024) [31]	Informal networks and grapevine communication	Fostering open and transparent formal communication channels. Cultivating trust-based relationships and ensuring adequate access to accurate information. Strategically prioritizing messages to mitigate information overload and actively identifying and dispelling false rumors without threatening informal group dynamics.
(French-Bravo et al., 2020) [32]	Cultivating staff buy-in for organizational initiatives	Utilizing a multimodal communication approach and leveraging objective data to substantiate the rationale for change. Acting as a strategic filter for system-level messaging to mitigate unit-level resistance. Promoting shared decision-making by explaining “the why” and employing active listening to address barriers.
(Gan, 2019) [33]	Alternative work arrangements (e.g., temporary, agency, or float nurses)	“Over-communicating” via redundant channels to ensure message saturation across shifts. Prioritizing face-to-face interactions to interpret nonverbal cues and establish “communication comfort accurately”. Tailoring mentoring resources and disciplinary feedback pathways according to the nurses’ employment status.
(Hartung & Miller, 2018) [34]	Rural healthcare settings and geographic isolation	Implementing redundant communication methods to circumvent unreliable technological infrastructure. Maximizing visibility, availability, and accessibility through regular face-to-face huddles. Adapting communication styles to generational contexts while maintaining a constructive and positive tone.
(Kämäräinen et al., 2022) [35]	Internal crisis communication (e.g., COVID-19 pandemic)	Delivering timely, regular, unequivocal, and evidence-based information. Fostering two-way interaction characterized by empathy, active listening, and emotional support to alleviate stress, mitigate rumors, and reduce uncertainty.
(Sharpp et al., 2019) [36]	e-Leadership and ICT integration	Cultivating a robust virtual “presence” and addressing the socio-emotional needs of geographically dispersed teams. Providing hands-on mentoring for new technological applications and mitigating information and email overload by establishing clear electronic communication norms.

Source: Authors’ own elaboration.

**Table 3:** Antecedents and Consequences of Nurse Managers’ Communication Competency.

Author & Year	Antecedents (Prerequisites and Moderators)	Consequences (Organizational and Clinical Outcomes)
(Khosravani et al., 2018) [30]	Higher ethical sensitivity among nurse managers.	Higher ethical sensitivity was associated with better communication skills, including listening, verbal communication, and feedback skills.
(Donohue-Porter et al., 2019) [37]	Application of the Leader-Member Exchange (LMX) theory, characterized by fair dyadic relationships built on trust, respect, and mutual obligation.	Significant augmentation of global job satisfaction and organizational commitment, fostering structural and psychological empowerment, and promoting retention of the nursing workforce.
(French-Bravo et al., 2020) [32]	Active managerial engagement, implementation of shared decision-making frameworks, and execution of a multimodal communication strategy leveraging objective data.	Cultivation of robust nurse buy-in for quality initiatives, enhanced Patients’ Experiences with Care (PEC), psychological safety for staff, and actualization of a patient-centered care culture.
(Hartung & Miller, 2018) [34]	Managerial visibility, accessibility, consistent contact, and congruent values aligned with the specific rural/isolated geographic subculture.	Workload optimization and efficiency, stabilization of the rural workforce, cultivation of cohesive and well-functioning interdisciplinary teams, and enhanced employee morale.
(Hola et al., 2020) [38]	Establishment of continuous lifelong learning programs, a structural formal setting of work, and proactive top management support.	Elevated employee engagement and productivity, augmented organizational identification and loyalty, reduced turnover intentions, and enhanced employer reputation.
(Hopkinson et al., 2021) [39]	Structural empowerment, provision of timely access to critical information, and dedicated leadership communication training.	Facilitation of psychological empowerment among staff nurses—specifically enhancing the dimensions of professional impact and self-determination—and stimulating followership and job satisfaction.
(Jankelova & Joniakova, 2021) [40]	Lower span of control (reduced number of direct subordinates), shorter/optimal management practice tenure, preventing stereotyping, and robust psychosocial work factors (organizational support).	Mitigation of occupational stress and emotional exhaustion, increased clinical teamwork, stabilization of the nursing workforce, and heightened perceived quality of healthcare by patients.
(Mohammadi et al., 2016) [41]	Implementation of targeted communication skills training, problem-solving, and continuous professional development.	Heightened clinical self-efficacy among nurses leads to enhanced performance, improved quality of patient care, and optimal health system functioning.

Source: Authors’ own elaboration.

**Table 4:** Empowering and Limiting Communication Behaviors Associated With Nurse Managers’ Communication Competency.

Author & Year	Empowering Behaviors (Positive)	Limiting Behaviors (Negative)
(French-Bravo et al., 2020) [32]	Facilitating shared decision-making and sharing accountability for both clinical successes and failures. Inquiring proactively about clinical barriers (asking “the why”) and acting as a translational filter for systemic directives to mitigate unit-level resistance. Validating nurses’ professional perspectives and fostering a psychologically safe environment.	Imposing top-down directives without soliciting clinical input or feedback. Adopting a punitive or punishing communicative stance during operational failures rather than utilizing a collaborative problem-solving approach.
(Hopkinson et al., 2019) [29]	Utilizing active listening by giving undivided attention without interruptions, and ensuring comprehensibility through clear instructions.	Exhibiting condescending or unprofessional manners and abruptly interrupting two-way conversations.
(Hopkinson et al., 2021) [39]	Fostering openness by soliciting input, expressing empathy by acknowledging emotions, and delivering timely, constructive feedback in private. Displaying positive nonverbal cues, such as a calm demeanor and friendly eye contact.	Providing negative feedback publicly or at the last minute, demonstrating dismissive nonverbal behaviors (e.g., rolling eyes, turning away), and relying on aggressive paralanguage such as yelling
(Kämäräinen et al., 2022) [35]	Providing timely, unequivocal, regular, and evidence-based informational updates during organizational crises. Demonstrating empathic interaction that actively alleviates organizational stress and mitigates clinical anxiety.	Disseminating ambiguous, false, or delayed information during critical events. Failing to address workplace uncertainties, thereby allowing the proliferation of organizational misinformation and informal rumors.

Source: Authors’ own elaboration.

**Table 5:** Moderators, Barriers, and Facilitators of Communication Effectiveness in Nurse Managers.

Author & Year	Factor Classification	Synthesized Impact on Communication Effectiveness
(Khosravani et al., 2018) [30]	Barrier/Facilitator	Crisis-induced psychological distress (e.g., fear, anxiety, uncertainty) and message ambiguity significantly impair cognitive information processing. To facilitate comprehension, leaders must mitigate these barriers by deploying simple, precise, and highly consistent messaging.
(Elsayed et al., 2024) [31]	Barrier/Facilitator	Unmanaged informal networks (the grapevine), the proliferation of rumors, and information overload act as profound organizational barriers. Providing transparent, formal communication channels and ensuring adequate access to verifiable information are critical facilitators in mitigating workplace misinformation.
(Gan, 2019) [33]	Moderator/Barrier	Alternative work arrangements (e.g., temporary, float, or agency staffing) significantly moderate the depth of communication. The transient nature of these roles hinders trust-building, prompting managers to allocate limited mentoring and communication resources primarily to permanent staff.
(Hartung & Miller, 2018) [34]	Barrier/Facilitator	Geographic isolation and unreliable technological infrastructure (e.g., limited cell tower coverage) pose significant communication barriers in rural clinical settings. These impediments are circumvented by implementing redundant, multimodal communication channels and frequent face-to-face huddles.
(Hopkinson et al., 2021) [39]	Barrier/Facilitator	Limiting behaviors—such as abrupt communication, dismissive nonverbal cues (e.g., eye-rolling), and delayed information dissemination—act as significant barriers to staff empowerment. Conversely, active listening, empathetic openness, and comprehensibility function as potent facilitators of effective leadership communication.
Jankelová & Joniaková (2021) [40]	Moderator	The manager’s span of control and tenure significantly moderate communication efficacy. A narrower span of control and a shorter management tenure strengthen the positive effects of communication skills on nurses’ job satisfaction, preventing managerial rigidity and stereotyping. Psychosocial work factors (e.g., organizational support) also strongly moderate this relationship.
(Luo et al., 2016) [42]	Barrier	Interprofessional hierarchy (e.g., friction when communicating with physicians) and generational differences with younger nurses pose substantial communication barriers. Furthermore, occupational stressors, including role ambiguity and role overload, precipitate emotional exhaustion and impede effective managerial interactions.
(Sharpp et al., 2019) [36]	Barrier/Facilitator	Information overload (“too much, too many”), device proliferation, and inadequate technological onboarding severely hinder effective e-Leadership and the maintenance of virtual relationships. Dedicated mentorship and comprehensive training on Information and Communication Technology (ICT) facilitate optimal digital integration.

Source: Authors’ own elaboration.

## Data Availability

No new data were created or analyzed in this study. Data sharing is not applicable to this article.

## Supplementary Materials

The following supporting information can be downloaded at: https://www.mdpi.com/article/10.3390/healthcare14172814/s1, File S1: Table S1. Article search syntax; Table S2. Main Characteristics of the Included Studies; Table S3. Quality Appraisal of Quantitative Studies; Table S4. Quality Appraisal of Qualitative Studies; Table S5. Risk of Bias of the Included Studies; Table S6. Conceptual Models and Assessment Tools Used to Examine Nurse Managers’ Communication Competency; Table S7. Allocation of Included Studies to the Review Syntheses; File S2: PRISMA 2020 checklist.

## References

[1] Lenssen E., Nagtegaal I., van Oostveen C., Sieben A., van Rijssen L., Weggelaar A.M. (2025). Exploring nurses’ leadership and resilience in a complex daily work environment: A qualitative study. BMC Nurs..

[2] Hedqvist A., Praetorius G., Ekstedt M., Lindberg C. (2025). Entangled in complexity: An ethnographic study of organizational adaptability and safe care transitions for patients with complex care needs. J. Adv. Nurs..

[3] Kraus S., Schiavone F., Pluzhnikova A., Invernizzi A.C. (2021). Digital transformation in healthcare: Analyzing the current state-of-research. J. Bus. Res..

[4] Warshawsky N.E., Cramer E., Grandfield E.M., Schlotzhauer A.E. (2022). The influence of nurse manager competency on practice environment, missed nursing care, and patient care quality: A cross-sectional study of nurse managers in U.S. hospitals. J. Nurs. Manag..

[5] Jordal K., Saltveit V., Tønnessen S. (2022). Nursing leadership and management in home care: A qualitative scoping review. J. Nurs. Manag..

[6] Alanazi N.H., Alshamlani Y., Baker O.G. (2023). The association between nurse managers’ transformational leadership and quality of patient care: A systematic review. Int. Nurs. Rev..

[7] Wang S., Tong J., Wang Y., Zhang D. (2022). A study on nurse manager competency model of tertiary general hospitals in China. Int. J. Environ. Res. Public Health.

[8] Ofei A.M.A., Paarima Y., Barnes T. (2020). Exploring the management competencies of nurse managers in the Greater Accra Region, Ghana. Int. J. Afr. Nurs. Sci..

[9] González-García A., Pinto-Carral A., Pérez-González S., Marqués-Sánchez P. (2021). Nurse managers’ competencies: A scoping review. J. Nurs. Manag..

[10] Billiau L., Taghon D., Duprez V., Eeckloo K., Malfait S. (2025). Instruments for measuring head nurses’ competencies in a hospital setting: A scoping review. BMC Nurs..

[11] Filomeno L., Forte D., Di Simone E., Di Muzio M., Tartaglini D., Lommi M., Ivziku D. (2024). Systematic review and psychometric properties analysis of first-, middle-, and top-level nurse manager’s core competencies instruments. J. Nurs. Manag..

[12] Pérez-González S., Marqués-Sánchez P., Pinto-Carral A., González-García A., Liébana-Presa C., Benavides C. (2024). Characteristics of leadership competency in nurse managers: A scoping review. J. Nurs. Manag..

[13] Arias J.M.V., Peres A.M., Nunes M.G.J., Rico F.M.E., Noreña-Peña A. (2025). Essential Competencies Required of Nurse Managers in Times of COVID-19: A scoping Review. Int. Nurs. Rev..

[14] Choi P.-P., Lee W.-M., Wong S.-S., Tiu M.-H. (2022). Competencies of nurse managers as predictors of staff nurses’ job satisfaction and turnover intention. Int. J. Environ. Res. Public Health.

[15] Fowler K.R., Robbins L.K., Lucero A. (2021). Nurse Manager Communication and Outcomes for Nursing: An Integrative Review. J. Nurs. Manag..

[16] Asgari N., Mohammadi E., Kazemnejad A., Navipour H. (2021). Psychometric properties of nursing manager communication competency questionnaire (CCCQ): An instrument design study. Ann. Glob. Health.

[17] Kiviniitty N., Kamau S., Mikkonen K., Hammarén M., Koskenranta M., Kuivila H., Kanste O. (2023). Nurse leaders’ perceptions of competence-based management of culturally and linguistically diverse nurses: A descriptive qualitative study. Nurs. Open.

[18] Kvist T., Seitovirta J., Nurmeksela A. (2022). Nursing leadership from crisis to postpandemic. J. Nurs. Manag..

[19] Simonovich S.D., Spurlark R.S., Badowski D., Krawczyk S., Soco C., Ponder T.N., Rhyner D., Waid R., Aquino E., Lattner C. (2021). Examining effective communication in nursing practice during COVID-19: A large-scale qualitative study. Int. Nurs. Rev..

[20] Manoppo I.A., Novieastari E., Handiyani H., Nuraini T. (2024). Nursing competency model for nurse manager in hospital: A scoping review. Healthc. Low-Resour. Settings.

[21] Soares S.F., Carvalho Moura E.C., Lopez V., Peres A.M. (2021). Professional nursing communication competence: Theoretical procedures for instrument development and pilot test. J. Nurs. Manag..

[22] Page M.J., McKenzie J.E., Bossuyt P.M., Boutron I., Hoffmann T.C., Mulrow C.D., Shamseer L., Tetzlaff J.M., Akl E.A., Brennan S.E. (2021). The PRISMA 2020 statement: An updated guideline for reporting systematic reviews. PLoS Med..

[23] Aromataris E., Munn Z. (2020). JBI Manual for Evidence Synthesis.

[24] Moola S., Munn Z., Tufanaru C., Aromataris E., Sears K., Sfetcu R., Currie M., Qureshi R., Mattis P., Lisy K., Aromataris E., Munn Z. (2020). Chapter 7: Systematic reviews of etiology and risk. JBI Manual for Evidence Synthesis.

[25] Lockwood C., Munn Z., Porritt K. (2015). Qualitative research synthesis. Int. J. Evid. Based Healthc..

[26] Peters M.D.J., Godfrey C.M., Khalil H., McInerney P., Parker D., Soares C.B. (2015). Guidance for conducting systematic scoping reviews. Int. J. Evid. Based Healthc..

[27] González-García A. (2019). Modelo de Competencias para la Gestora Enfermera. Ph.D. Thesis.

[28] Campbell M., McKenzie J.E., Sowden A., Katikireddi S.V., Brennan S.E., Ellis S., Hartmann-Boyce J., Ryan R., Shepperd S., Thomas J. (2020). Synthesis without meta-analysis (SWiM) in systematic reviews: Reporting guideline. BMJ.

[29] Hopkinson S.G., Oblea P., Napier C., Lasiowski J., Trego L.L. (2019). Identifying the constructs of empowering nurse leader communication through an instrument development process. J. Nurs. Manag..

[30] Khosravani M., Borhani F., Loghmani L., Mohsenpour M. (2018). Ethical sensitivity relationship with communication skills in Iranian nursing managers. Int. J. Pharm. Res..

[31] Elsayed A.M., Elsayed K.A., Ghadery S.H., Abou Ramadan A.H. (2024). Assess nurse managers’ management of grapevine communication among nursing staff. Tanta Sci. Nurs. J..

[32] French-Bravo M., Nelson-Brantley H.V., Williams K., Ford D.J., Manos L., Veazey Brooks J. (2020). Exploring nurses’ perceptions of nurse managers’ communicative relationships that encourage nurses’ decisions to buy-in to initiatives that enhance patients’ experiences with care. J. Nurs. Manag..

[33] Gan I. (2019). How do nurses’ work arrangements influence nurse managers’ communication? A qualitative study. J. Nurs. Manag..

[34] Hartung S.Q., Miller M. (2018). Rural nurse managers’ perspectives into better communication practices. J. Community Health Nurs..

[35] Kämäräinen P.M., Nurmeksela A., Kvist T. (2022). A cross-sectional study of nurses’ perceptions of nurse leaders’ internal crisis communication during the COVID-19 pandemic. J. Nurs. Manag..

[36] Sharpp T.J., Lovelace K., Cowan L.D., Baker D. (2019). Perspectives of nurse managers on information communication technology and e-leadership. J. Nurs. Manag..

[37] Donohue-Porter P., Eckardt P., Prottas D., Rondello K.C., Silberstang J. (2019). A bridge to leadership communication success: Impact of leader-member exchange on nursing administrative relationships. Nurse Lead..

[38] Holá J., Moravcová M., Hlaváčková E. (2020). Communication competency: The topic of lifelong learning for nurse managers in hospitals. Kontakt.

[39] Hopkinson S.G., Glaser D., Napier C., Trego L.L. (2021). Developing an instrument to assess empowering nurse leader communication behaviours. J. Nurs. Manag..

[40] Jankelová N., Joniaková Z. (2021). Communication skills and transformational leadership style of first-line nurse managers in relation to job satisfaction of nurses and moderators of this relationship. Healthcare.

[41] Mohammadi M., Mamikhani J., Roshani D., Fatehi F., Asefzadeh S. (2016). Examining the relationship between self-efficacy of nurses with communication skills of head nurses. J. Chem. Pharm. Sci..

[42] Luo W.Y., Shen N.P., Lou J.H., He P.P., Sun J.W. (2016). Exploring competencies: A qualitative study of Chinese nurse managers. J. Nurs. Manag..

[43] Al-Thawabiya A., Singh K., Al-Lenjawi B.A., Alomari A. (2023). Leadership styles and transformational leadership skills among nurse leaders in Qatar, a cross-sectional study. Nurs. Open.

[44] Zusman N., Scheinberg Andrews C., Kaslin V., Kienski Woloski Wruble A.C. (2025). Creating a supportive work environment: A cognitive behavioral approach for nurse leaders. Nurs. Rep..

[45] Kwame A., Petrucka P.M. (2021). A literature-based study of patient-centered care and communication in nurse-patient interactions: Barriers, facilitators, and the way forward. BMC Nurs..

[46] Starc J., Neuberg M., Erjavec K. (2019). Nurses’ satisfaction with the use of communication channels by their managers in Croatia and Slovenia. Manag. J. Contemp. Manag. Issues.

[47] Varga A.I., Spehar I., Veggeland F., Skirbekk H. (2025). Factors influencing trust among colleagues in hospital settings: A systematic review. BMC Health Serv. Res..

[48] Notarnicola I., Duka B., Lommi M., Grosha E., De Maria M., Iacorossi L., Mastroianni C., Ivziku D., Rocco G., Stievano A. (2024). Transformational leadership and its impact on job satisfaction and personal mastery for nursing leaders in healthcare organizations. Nurs. Rep..

[49] Ahlqvist A., Nurmeksela A., Kvist T. (2023). The COVID-19 pandemic challenged nurse managers’ daily leadership work: A qualitative study. J. Nurs. Manag..

[50] Musitia P., Boga M., Oluoch D., Haaland A., Nzinga J., English M., Molyneux S. (2025). Strengthening respectful communication with patients and colleagues in neonatal units—Developing and evaluating a communication and emotional competence training for nurse managers in Kenya. Wellcome Open Res..

[51] Ystaas L.M.K., Nikitara M., Ghobrial S., Latzourakis E., Polychronis G., Constantinou C.S. (2023). The impact of transformational leadership in the nursing work environment and patients’ outcomes: A systematic review. Nurs. Rep..

[52] Abou Hashish E.A., Khattab S.M.A.K., Mohammad H.F., Elliethey N.S. (2025). Exploring the impact of leadership, organizational support, and knowledge management in evidence-based practice implementation among nurse managers through SEM. Worldviews Evid. Based Nurs..

[53] Cho H., Steege L.M., Arsenault Knudsen É.N. (2023). Psychological safety, communication openness, nurse job outcomes, and patient safety in hospital nurses. Res. Nurs. Health.

[54] O’DOnovan R., Rogers L., Khurshid Z., De Brún A., Nicholson E., O’SHea M., Ward M., McAuliffe E. (2021). A systematic review exploring the impact of focal leader behaviours on health care team performance. J. Nurs. Manag..

[55] Ip E., Srivastava R., Lentz L., Jasinoski S., Anderson G.S. (2025). Antecedents of workplace psychological safety in public safety and frontline healthcare: A scoping review. Int. J. Environ. Res. Public Health.

[56] Stephen Ekpenyong M., Nyashanu M., Ossey-Nweze C., Serrant L. (2021). Exploring the perceptions of dignity among patients and nurses in hospital and community settings: An integrative review. J. Res. Nurs..

[57] Alsadaan N., Alqahtani M. (2024). Toxic leadership in emergency nurses: Assessing abusive supervision and its team-level impacts on conflict management and organizational commitment. J. Nurs. Manag..

[58] Lee W., Jang I. (2023). Effect of nurses’ professionalism, work environment, and communication with health professionals on patient safety culture (AHRQ 2.0): A cross-sectional multicenter study. J. Nurs. Manag..

