# Supplementary material for: Characteristics of Communication Competency in Nurse Managers: A Systematic Review
Source: Healthcare (Basel). 2026 Sep 2;14(17):2814. doi: 10.3390/healthcare14172814 (PMC13566562; doi:10.3390/healthcare14172814)
Supplement: Supplementary file 1 [file healthcare-14-02814-s001.zip › healthcare-4472272_Supplementary_File_S1.pdf]

Systematic Review

Characteristics of Communication Competency in Nurse Managers: A Systematic Review

Supplementary File S1

Table S1. Article search syntax

**Database**	2016-2026 Search Terms
**WOS**	TI=(communication* OR "communication skill*" OR "interpersonal communication" OR "leadership communication") AND TI=("nurs* manag*" OR "nurs* supervis*" OR "nursing program manager" OR "nurse unit manager" OR "chief nurse executive" OR "nurs* administrat*" OR "director of nurs*" OR "head nurs*" OR "frontline manager" OR "nurs* director" OR "nurs* executiv*") AND TS=("communication*" OR "communication skill*" OR "interpersonal communication" OR "leadership communication") AND TS=("nurs*manag*" OR "nurs* supervis*" OR "nursing program manager" OR "nurse unit manager" OR "chief nurse executive" OR "nurs* administrat*" OR "director of nurs*" OR "head nurs*" OR "frontline manager" OR "nurs* director" OR "nurs* executiv*")
**SCOPUS**	TITLE-ABS-KEY ("communication*" OR "communication skill*" OR "interpersonal communication" OR "leadership communication") AND TITLE-ABS-KEY ("nurs* manag*" OR "nurs* supervis*" OR "nursing program manager" OR "nurse unit manager" OR "chief nurse executive" OR "nurs* administrat*" OR "director of nurs*" OR "head nurs*" OR "frontline manager" OR "nurs* director" OR "nurs* executiv*")
**PUBMED**	("Communication"[MeSH Terms] OR "Communication"[All Fields] OR "Communication Skills"[MeSH Terms] OR "communication skill*"[All Fields] OR "interpersonal communication"[All Fields] OR "leadership communication"[All Fields]) AND ("Nurse Administrators"[MeSH Terms] OR "Nursing, Supervisory"[MeSH Terms] OR "Nurse Managers"[All Fields] OR "nurs* manag*"[All Fields] OR "nurs* supervis*"[All Fields] OR "nursing program manager"[All Fields] OR "nurse unit manager"[All Fields] OR "chief nurse executive"[All Fields] OR "nurs* administrat*"[All Fields] OR "director of nurs*"[All Fields] OR "head nurs*"[All Fields] OR "frontline manager"[All Fields] OR "nurs* director"[All Fields] OR "nurs* executiv*"[All Fields])
**CINAHL**	(MH "Communication" OR MH "Communication Skills" OR MH "Interpersonal Communication" OR "communication*" OR "communication skill*" OR "interpersonal communication" OR "leadership communication") AND (MH "Nurse Administrators" OR MH "Nurse Managers" OR MH "Supervisory, Nursing" OR "nurs* manag*" OR "nurs* supervis*" OR "nursing program manager" OR "nurse unit manager" OR "chief nurse executive" OR "nurs* administrat*" OR "director of nurs*" OR "head nurs*" OR "frontline manager" OR "nurs* director" OR "nurs* executiv*")

Table S2. Main Characteristics of the Included Studies

Author & Year	Country	Study Design	Methodology	Participants	Variables	Implications for Nurse Managers	Main Findings	Limitations
**(Asgari et al., 2021) [1]**	Iran	Methodological	Factor analysis	300 nurses, 30 experts	NMCCQ scale	Use scale for manager selection/appointment.	Created a valid, reliable 56-item questionnaire across 5 dimensions.	Cross-sectional validation.
**(Donohue-Porter et al., 2019) [2]**	USA	Correlational	Questionnaires	206 nurses	Leader-member exchange (LMX)	Train managers in relational skills.	Better leader-member relationship significantly boosts job satisfaction.	Low response; concurrent surveys.
**(Elsayed et al., 2024) [3]**	Egypt	Correlational	Questionnaires	35 managers, 260 nurses	Grapevine, communication management	Foster open channels to prevent rumors.	Positive correlation between informal channels (rumors) and management handling.	Cross-sectional; local sample.
**(French-Bravo et al., 2020) [4]**	USA	Qualitative	Semi-structured interviews	15 nurses	Communication, buy-in	Use multimodal approach and share decision-making.	Strong communication and mutual support secure staff buy-in for projects.	Selection bias; Lean context.
**(Gan, 2019) [5]**	USA	Qualitative	Grounded theory	26 managers	Contract type, mentoring	Standardize mentoring for contingent staff.	Mentoring prioritizes permanent staff; communication varies by contract.	Assesses only managers◎ views.
**(Hartung & Miller, 2018) [6]**	USA	Qualitative	Grounded theory	9 managers	Rural setting, communication	Use brief huddles and message redundancy.	Face-to-face preferred in rural areas, but distance requires varied channels.	Tiny sample; rural context.
**(Holá et al., 2020) [7]**	Czech Rep.	Cross-sectional	Quant. survey	1,564 staff, 102 managers	Satisfaction, communication skills	Include communication in lifelong learning.	Huge gap between perceived importance of communication and actual skills.	Self-reported; non-parametric analysis.
**(Hopkinson et al., 2019) [8]**	USA	Methodological	Scale development	16 (focus), 42 (pilot)	Empowering communication	Use constructs to assess leadership.	Identified 8 key constructs (listening, empathy, tone, openness, etc.).	Strict military setting.

**(Hopkinson et al., 2021) [9]**	USA	Cross-sectional	Factor analysis	240 nurses	Empowering/limiting communication	Train in communication to empower the team.	Validated 2 clear factors: empowering vs. limiting communication behaviors.	Low response rate; biases.
**(Jankelova & Joniakova, 2021) [10]**	Slovakia	Cross-sectional	Questionnaires, regression	132 managers, 1,120 nurses	Satisfaction, leadership, control span, practice	Avoid substituting management training with mere years of practice.	Transformational leadership and communication drastically improve satisfaction.	Focused sample; cross-sectional.
**(Kämäräinen et al., 2022) [11]**	Finland	Cross-sectional	Online survey	204 nurses	Crisis communication, COVID-19	Adapt info to unit type (ICU vs. ward).	Communication was timely but lacked managerial interaction and empathy.	Young sample; via social media.
**(Khosravani et al., 2018) [12]**	Iran	Cross-sectional	Questionnaires	60 managers	Ethical sensitivity, communication	Strengthen ethics for effective communication.	High ethical sensitivity relates to better communication skills.	Small sample; cultural factors.
**(Luo et al., 2016) [13]**	China	Qualitative	Phenomenology	12 managers	Competencies, role transition	Formal training in stress management is required.	Transition has 4 phases; communication is the hardest competency to master.	Very small sample; one province.
**(Mohammadi et al., 2016) [14]**	Iran	Cross-sectional	Questionnaires	51 managers, 153 nurses	Communication, self-efficacy	Plan communication workshops and incentives.	Higher manager communication skills increase nurse self-efficacy.	Local teaching hospitals only.
**(Sharpp et al., 2019) [15]**	USA	Qualitative	Interviews, focus groups	16 managers	e-Leadership, ICT use	Mentor and structure tech onboarding.	ICTs cause overload and stress due to excessive emails and lack of training.	Single hospital; novice managers.

Table S3. Application of the JBI Critical Appraisal Checklist for Analytical Cross-Sectional Studies

Author & Year	Q1	Q2	Q3	Q4	Q5	Q6	Q7	Q8	Total score	Share of answers yes (%)
**(Asgari et al., 2021) [1]**	Yes	Yes	No	No	No	No	Yes	Yes	4/8	50%
**(Donohue-Porter et al., 2019) [2]**	Yes	Yes	Yes	Yes	Yes	Yes	Yes	Yes	8/8	100%
**(Elsayed et al., 2024) [3]**	Yes	Yes	Yes	Yes	Yes	No	Yes	Yes	7/8	87.5%
**(Hola et al., 2020) [7]**	Yes	Yes	Yes	Yes	Yes	No	Yes	Yes	7/8	87.5%
**(Hopkinson et al., 2019) [8]**	Yes	Yes	No	No	No	No	Yes	Yes	4/8	50%
**(Hopkinson et al., 2021) [9]**	Yes	Yes	Yes	Yes	Yes	No	Yes	Yes	7/8	87.5%
**(Jankelova & Joniakova, 2021) [10]**	Yes	Yes	Yes	Yes	Yes	Yes	Yes	Yes	8/8	100%
**(Kämäräinen et al., 2022) [11]**	Yes	Yes	Yes	Yes	Yes	No	Yes	Yes	7/8	87.5%
**(Khosravani et al., 2018) [12]**	Yes	Yes	Yes	Yes	Yes	No	Yes	Yes	7/8	87.5%
**(Mohammadi et al., 2016) [14]**	Yes	Yes	Yes	Yes	Yes	No	Yes	Yes	7/8	87.5%

Note: The adherence percentage was calculated as the number of "Yes" responses divided by the total number of checklist items and multiplied by 100. "No" and "Unclear" responses were scored as zero.

Table S4. Application of the JBI Critical Appraisal Checklist for Qualitative Research

Author & Year	Q1	Q2	Q3	Q4	Q5	Q6	Q7	Q8	Q9	Q10	Total score	Share of answers yes (%)
**(French-Bravo et al., 2020) [4]**	Yes	Yes	Yes	Yes	Yes	No	Yes	Yes	Yes	Yes	9/10	90%
**(Gan, 2019) [5]**	Yes	Yes	Yes	Yes	Yes	Yes	Yes	Yes	Yes	Yes	10/10	100%
**(Hartung & Miller, 2018) [6]**	Yes	Yes	Yes	Yes	Yes	No	Unclear	Yes	Unclear	Yes	7/10	70%
**(Luo et al., 2016) [13]**	Yes	Yes	Yes	Yes	Yes	No	Unclear	Yes	Yes	Yes	8/10	80%
**(Sharpp et al., 2019) [15]**	Yes	Yes	Yes	Yes	Yes	No	Yes	Yes	Yes	Yes	9/10	90%

Note: The adherence percentage was calculated as the number of "Yes" responses divided by the total number of checklist items and multiplied by 100. "No" and "Unclear" responses were scored as zero.

Table S5. Risk of Bias of the Included Studies

Author & Year	Study Design	Sample Size	Bias Evaluation	Risk of Bias
**(Mohammadi et al., 2016) [14]**	Analytical cross-sectional	204	Standardized questionnaires utilized, but data analysis relied on descriptive and bivariate statistics without multivariate control for confounding factors.	Moderate
**(Asgari et al., 2021) [1]**	Instrument design (Mixed methods)	315	High methodological rigor using exploratory factor analysis and content validity indices, though utilizing a convenience sampling approach.	Low
**(Donohue-Porter et al., 2019) [2]**	Cross-sectional descriptive	206	Strong multilevel mixed-modeling accounts for clustering, though the study is limited by a low response rate (34%) and convenience sampling.	Moderate

**(Elsayed et al., 2024) [3]**	Descriptive correlational, cross-sectional	295	Proportional random sampling implemented with valid tools, but the statistical approach lacks multivariate regression models to control for demographic confounders.	Moderate
**(French-Bravo et al., 2020) [4]**	Exploratory qualitative descriptive	15	High methodological quality established through data triangulation, member checking, and robust peer debriefing processes.	Low
**(Gan, 2019) [5]**	Qualitative grounded theory	26	High qualitative rigor demonstrating theoretical sampling, constant comparative analysis, and achievement of theoretical saturation.	Low
**(Hartung & Miller, 2018) [6]**	Qualitative grounded theory	9	Good practices in interviews and coding using QDA software, though findings are limited by a small sample size and voluntary recruitment.	Moderate
**(Hola et al., 2020) [7]**	Quantitative cross-sectional survey	1,666	Large sample size with validated tools, but limited control of confounding variables due to a reliance on non-parametric correlational tests.	Moderate
**(Hopkinson et al., 2019) [8]**	Mixed methods (Instrument development)	58	Stringent instrument development protocol utilizing focus groups, though the quantitative pilot testing is limited by a very small sample size.	Moderate
**(Hopkinson et al., 2021) [9]**	Cross-sectional (Instrument validation)	240	Robust exploratory and confirmatory factor analyses, but highly susceptible to selection bias due to a low 13% response rate.	Moderate
**(Jankelova & Joniakova, 2021) [10]**	Cross-sectional survey	132	Excellent control of variables using hierarchical moderated regression, effectively mitigating organizational and demographic confounding factors.	Low
**(Kämäräinen et al., 2022) [11]**	Cross-sectional survey	204	Large dataset with reliable instrumentation, though social media convenience sampling introduces potential selection bias.	Moderate
**(Khosravani et al., 2018) [12]**	Cross-sectional	60	Census sampling utilized with valid tools; however, bivariate analyses limit the robust control of confounding variables.	Moderate
**(Luo et al., 2016) [13]**	Qualitative descriptive phenomenology	12	In-depth phenomenological approach with an audit trail, but sample size is limited and restricted to a single geographical province.	Moderate
**(Sharpp et al., 2019) [15]**	Qualitative exploratory descriptive	16	Strong methodological rigor demonstrated through method triangulation, member checking, and peer collaboration.	Low

Note: Risk-of-bias categories were based on a qualitative domain-based judgment and were not derived from numerical adherence-score thresholds.

Table S6. Conceptual Models and Assessment Tools Used to Examine Nurse Managers' Communication Competency

**Author & Year**	**Model / Tool**	**Synthesized Dimensions and Evaluated Constructs**

**(Asgari et al., 2021) [1]**	Nursing Manager Communication Competency Questionnaire (NMCCQ)	Non-verbal communication skills, negotiation, reporting, communication ethics, and the application of communication knowledge.
**(Donohue-Porter et al., 2019) [2]**	Leader-Member Exchange (LMX) Theory	Quality of dyadic relationships characterized by respect, trust, and obligation, which fosters autonomy, open communication, and participation in knowledge transfer.
**(Elsayed et al., 2024) [3]**	Grapevine Communication Management Questionnaire	Providing open channels of communication, creating trust-relationships, providing adequate access to information, eliminating information overload, picking up and dispelling false rumors, and understanding/managing informal groups.
**(Hola et al., 2020) [7]**	Dewhurst and FitzPatrick’s Communication Competencies Model	12 core competencies: Building effective relationships, business focus, consultation and coaching, cross-functional awareness, craft (writing and design), developing other communicators, innovation and creativity, listening, making it happen, planning, specialist knowledge, and vision and standards.
**(Hopkinson et al., 2019) [8]**	Nurse Leader Communication Assessment	Empowering and limiting behaviors evaluated through eight constructs: Comprehensibility, listening, openness, feedback, empathy, nonverbal communication, paralanguage, and manner.
**(Hopkinson et al., 2021) [9]**	Nurse Leader Communication Assessment (final validated version)	Two-factor structure comprising 20 positive items assessing empowering communication behaviors and 15 negative items assessing limiting communication behaviors.
**(Jankelova & Joniakova, 2021) [10]**	Multifactor Leadership Questionnaire (MLQ) for Transformational Leadership (TFL)	Intellectual stimulation, inspirational motivation, idealized impact, and individual approach.
**(Kämäräinen et al., 2022) [11]**	Internal Crisis Communication Questionnaire	Interaction, content, timeliness, and false communication (rumors or misinformation).
**(Khosravani et al., 2018) [12]**	Barton’s Communication Skills Questionnaire	Divided into three main components: Listening skills, verbal communication, and feedback skills.
**(Luo et al., 2016) [13]**	Iceberg Model (Spencer & Spencer)	Hidden characteristics (self-concept, traits, and motives) and surface characteristics (knowledge and skills).
**(Mohammadi et al., 2016) [14]**	Queendom Communication Skills Inventory	Ability to receive and send messages, emotional control, listening, insight into the communication process, and communication combined with certainty.
**(Sharpp et al., 2019) [15]**	e-Leadership Conceptual Framework	Establishing a virtual "presence," tending to the social-emotional aspects of the team,

	establishing communication norms, and using technological tools in a timely way.

Table S7. Allocation of Included Studies to the Review Syntheses

Study	Syntheses to which the study contributed
**(Asgari et al., 2021) [1]**	Characteristics; conceptual models and assessment tools
**(Donohue-Porter et al., 2019) [2]**	Characteristics; antecedents and consequences; conceptual models and assessment tools
**(Elsayed et al., 2024) [3]**	Characteristics; context-specific strategies; moderators, barriers, and facilitators; conceptual models and assessment tools
**(French-Bravo et al., 2020) [4]**	Characteristics; context-specific strategies; antecedents and consequences; empowering and limiting behaviors
**(Gan, 2019) [5]**	Characteristics; context-specific strategies; moderators, barriers, and facilitators
**(Hartung & Miller, 2018) [6]**	Characteristics; context-specific strategies; antecedents and consequences; moderators, barriers, and facilitators
**(Hola et al., 2020) [7]**	Characteristics; antecedents and consequences; conceptual models and assessment tools
**(Hopkinson et al., 2019) [8]**	Characteristics; empowering and limiting behaviors; conceptual models and assessment tools
**(Hopkinson et al., 2021) [9]**	Characteristics; antecedents and consequences; empowering and limiting behaviors; moderators, barriers, and facilitators; assessment tools
**(Jankelova & Joniakova, 2021) [10]**	Characteristics; antecedents and consequences; moderators, barriers, and facilitators; conceptual models and assessment tools
**(Kämäräinen et al., 2022) [11]**	Characteristics; context-specific strategies; empowering and limiting behaviors; conceptual models and assessment tools
**(Khosravani et al., 2018) [12]**	Characteristics; antecedents and consequences; conceptual models and assessment tools
**(Luo et al., 2016) [13]**	Characteristics; moderators, barriers, and facilitators; conceptual models and assessment tools; developmental phases
**(Mohammadi et al., 2016) [14]**	Characteristics; antecedents and consequences; conceptual models and assessment tools
**(Sharpp et al., 2019) [15]**	Characteristics; context-specific strategies; moderators, barriers, and facilitators; conceptual models and assessment tools

References

1. Asgari, N.; Mohammadi, E.; Kazemnejad, A.; Navipour, H. Psychometric properties of nursing manager communication competency questionnaire (CCCQ): An instrument design study. *Ann. Glob. Health* **2021**, *87*, 25. https://doi.org/10.5334/aogh.3272.
2. Donohue-Porter, P.; Eckardt, P.; Prottas, D.; Rondello, K.C.; Silberstang, J. A bridge to leadership communication success: Impact of leader-member exchange on nursing administrative relationships. *Nurse Lead.* **2019**, *17*, 546–551. https://doi.org/10.1016/j.mnl.2019.02.006.
3. Elsayed, A.M.; Elsayed, K.A.; Ghadery, S.H.; Abou Ramadan, A.H. Assess nurse managers' management of grapevine communication among nursing staff. *Tanta Sci. Nurs. J.* **2024**, *34*, 196–211. https://doi.org/10.21608/tsnj.2024.375864.

4. French-Bravo, M.; Nelson-Brantley, H.V.; Williams, K.; Ford, D.J.; Manos, L.; Veazey Brooks, J. Exploring nurses' perceptions of nurse managers' communicative relationships that encourage nurses' decisions to buy-in to initiatives that enhance patients' experiences with care. *J. Nurs. Manag.* **2020**, *28*, 567–576. https://doi.org/10.1111/jonm.12958.
5. Gan, I. How do nurses' work arrangements influence nurse managers' communication? A qualitative study. *J. Nurs. Manag.* **2019**, *27*, 1366–1373. https://doi.org/10.1111/jonm.12817.
6. Hartung, S.Q.; Miller, M. Rural nurse managers' perspectives into better communication practices. *J. Community Health Nurs.* **2018**, *35*, 1–11. https://doi.org/10.1080/07370016.2018.1404829.
7. Holá, J.; Moravcová, M.; Hlaváčková, E. Communication competency: The topic of lifelong learning for nurse managers in hospitals. *Kontakt* **2020**, *22*, 33–39. https://doi.org/10.32725/kont.2019.053.
8. Hopkinson, S.G.; Oblea, P.; Napier, C.; Lasiowski, J.; Trego, L.L. Identifying the constructs of empowering nurse leader communication through an instrument development process. *J. Nurs. Manag.* **2019**, *27*, 722–731. https://doi.org/10.1111/jonm.12729.
9. Hopkinson, S.G.; Glaser, D.; Napier, C.; Trego, L.L. Developing an instrument to assess empowering nurse leader communication behaviours. *J. Nurs. Manag.* **2021**, *29*, 2037–2046. https://doi.org/10.1111/jonm.13340.
10. Jankelová, N.; Joniaková, Z. Communication skills and transformational leadership style of first-line nurse managers in relation to job satisfaction of nurses and moderators of this relationship. *Healthcare* **2021**, *9*, 346. https://doi.org/10.3390/healthcare9030346.
11. Kämäräinen, P.M.; Nurmeksela, A.; Kvist, T. A cross-sectional study of nurses' perceptions of nurse leaders' internal crisis communication during the COVID-19 pandemic. *J. Nurs. Manag.* **2022**, *30*, 2503–2513. https://doi.org/10.1111/jonm.13707.
12. Khosravani, M.; Borhani, F.; Loghmani, L.; Mohsenpour, M. Ethical sensitivity relationship with communication skills in Iranian nursing managers. *Int. J. Pharm. Res.* **2018**, *10*, 143–147. https://doi.org/10.31838/ijpr/2018.10.03.064.
13. Luo, W.Y.; Shen, N.P.; Lou, J.H.; He, P.P.; Sun, J.W. Exploring competencies: A qualitative study of Chinese nurse managers. *J. Nurs. Manag.* **2016**, *24*, E87–E94. https://doi.org/10.1111/jonm.12295.
14. Mohammadi, M.; Mamikhani, J.; Roshani, D.; Fatehi, F.; Asefzadeh, S. Examining the relationship between self-efficacy of nurses with communication skills of head nurses. *J. Chem. Pharm. Sci.* **2016**, *9*, 1386–1391. Available online: https://www.scopus.com/inward/record.uri?eid=2-s2.0-84988908428&partnerID=40&md5=9c3577e0f7b3085fe5e4a1f7456c2a21 (accessed on 25 June 2026).
15. Sharpp, T.J.; Lovelace, K.; Cowan, L.D.; Baker, D. Perspectives of nurse managers on information communication technology and e-leadership. *J. Nurs. Manag.* **2019**, *27*, 1554–1562. https://doi.org/10.1111/jonm.12845.
